# Long-term family outcomes in grade 2 IDH-mutated glioma patients treated with awake-guided surgery: Biological, professional, and therapeutic interactions

**DOI:** 10.1093/noajnl/vdaf102

**Published:** 2025-05-20

**Authors:** Sam Ng, Hugues Duffau

**Affiliations:** Institute of Functional Genomics, University of Montpellier, INSERM, CNRS, Team “Plasticity of Central Nervous System, Stem Cells and Glial Tumors,” National Institute for Health and Medical Research (INSERM), U1191 Laboratory, Montpellier, France; Department of Neurosurgery, Gui de Chauliac Hospital, Montpellier University Medical Center, Montpellier, France; Institute of Functional Genomics, University of Montpellier, INSERM, CNRS, Team “Plasticity of Central Nervous System, Stem Cells and Glial Tumors,” National Institute for Health and Medical Research (INSERM), U1191 Laboratory, Montpellier, France; Department of Neurosurgery, Gui de Chauliac Hospital, Montpellier University Medical Center, Montpellier, France

**Keywords:** awake surgery, family status, marital status, WHO grade 2 gliomas

## Abstract

**Background:**

Maintaining quality of life is a priority of the oncological management in IDH-mutated low-grade gliomas (IDHm-LGGs). Yet, family outcomes have received limited attention. This study aims to provide an overview of long-term family outcomes in IDHm-LGG patients.

**Methods:**

We retrospectively studied a consecutive cohort of IDHm-LGGs treated with awake functional-based resection (AwFR). The main outcomes were union/marriage, separation/divorce, and parenthood before/after surgery. We tested interactions with overall survival (OS), histomolecular data, therapeutics, occupations, and tumor locations.

**Results:**

A total of 538 patients were included (median age: 36 years [IQR: 30–44], 1p19q-codeletion: 237 [44.1%], median follow-up: 7.7 years [95% CI: 7.1–8.3]). Unions/marriages were observed in 374 (69.5%) patients preoperatively and were maintained in 329(61.2%) patients. Separations/divorces were observed in 41 (7.8%) patients. A total of 44 (8.2%) patients had new child/children, and 48 (9.1%) had new unions/marriages. Family status was stable in 399 (74.2%) patients. Prolonged single status was predicted by age (OR: 0.94, 95% CI: 0.91–0.98), female gender (OR: 0.48, 95% CI: 0.25–0.91), and previous child/children (OR: 0.23, 95% CI: 0.11–0.45). Total resection predicted new union/marriage and/or new child/children (OR: 2.59, 95% CI: 1.04–7.10). Occupational skill level (OR: 0.27, 95% CI: 0.05–0.95) predicted stable union/marriage, while previous parenthood predicted separation/divorce (OR: 2.73, 95% CI: 1.07–7.83). Lesion-symptom mapping revealed that right pars orbitalis/triangularis infiltrations (Liebermeister measures, *p*_corrected_ < 0.05, 10 000 permutations) were associated with separation/divorce. Family status did not impact OS, as assessed by multivariable proportional hazard and propensity matching-based survival analyses.

**Conclusions:**

This study offers a unique overview of the long-term family outcomes in IDHm-LGG patients. Critical interactions between familial, socio-professional, and oncological factors were highlighted. Despite the onset of the disease and the surgical approach, most patients had a stable family status.

**Key Points**

- Most IDHm-LGG patients treated with AwFR had a stable family status/continued to have family projects.
- Interactions between familial, professional, biological, and therapeutic factors were highlighted.
- Family status does not have an impact on OS.

**Importance of the Study**

Maintaining quality of life is a priority of the oncological management in IDHm-LGGs, but available data regarding long-term family outcomes, a major aspect of quality of life, are limited or missing. For the first time, in a large series of 538 molecularly defined IDHm-LGGs systematically operated with awake cognitive mapping, long-term family outcomes were investigated over an extended follow-up. Most patients presented with stable unions/marriages or new projects of parenthood despite the onset of the disease. Prolonged single status was predicted by age, female gender, and having a previous child/children. Medium occupational skill levels prevented separation/divorce. Total resections of FLAIR MRI signal predicted new union/marriage or new child/children in single patients, while tumors located in the right inferior frontal gyrus favored separation/divorce. Besides, family status did not have an impact on OS. This study offers a unique overview of interactions between familial, socio-professional, and oncological factors in patients with IDHm-LGG.

WHO grade 2 isocitrate dehydrogenase (IDH)-mutated low-grade gliomas (IDHm-LGGs)^1^ are diffuse neoplasms that constantly migrate within the brain and, if left untreated, ultimately progress to a higher grade of malignancy.^2^ IDHm-LGGs generally occur in young adult patients^3^ with active lives at diagnosis. Life expectancy may now exceed 2 decades, with a strong impact of histomolecular markers and surgical extent of resection.^4–7^ In this context, maintaining long-term quality of life has become a priority of oncological strategies and can be particularly challenging given the potential impact of the disease and the therapeutics themselves on short- and long-term neurological status.^7–9^ Overall, data suggest that well-being among patients and their caregivers may be significantly affected by the onset of the disease,^10^ together with their working abilities.^11^ However, contrasted results were highlighted by recent surgical cohort studies indicating that patients eligible for awake functional-based resection (AwFR) can display a high rate of preservation of cognitive functions,^12^ long-term preservation of overall autonomy,^13^ and a high rate of return to work^14^ in the year following surgery.

Besides, family outcomes in patients with IDHm-LGGs, a pivotal aspect of quality of life, are still underexplored. Yet, patients with brain tumors may suffer from psychological vulnerability and social frailties.^15^ Importantly, tumor-induced neurological incapacities, behavioral modifications^16^ or emotional recognition disorders,^17^ may further provoke social isolation and family breakdown.^18^ Another example of how the disease may affect patients’ family plans can be illustrated by the challenges faced by women desiring children, given the fact that pregnancy is highly suspected to have a pejorative impact on tumor growth^19^ and overall survival (OS).^20^

In addition, investigating the prognosis impact of patients’ family status may be of particular interest since it was suggested that marital status may have an independent effect on survival in patients with cancer in general^21^ and in patients with high-grade gliomas in particular.^22,23^ Such onco-functional measures have raised limited attention in current literature, although they may be essential (1) to inform patients and their family members before any treatment project, (2) to adapt personalized oncological strategies in view of patients’ family projects in order to optimize quality of life^24^ (eg, by defining the surgical strategy, or by accompanying a pregnancy project for example), and (3) to develop suitable rehabilitation or social support in family environments at risk of negative outcomes.

In this study, we aimed to measure the long-term family outcomes in patients with IDHm-LGGs who were initially treated with AwFR, over an extended period of follow-up. To this end, we leveraged a monocentric cohort of 538 IDHm-LGGs, included over a period of 26 years, to capture the interactions between long-term evolving family factors (stable union/marriage, having child/children, separation/divorce, new union/marriage), clinical, histomolecular, radiological, socio-professional, therapeutic factors and overall survival (OS).

## Methods

### Study Design and Participants

In this retrospective cohort analysis, participants were extracted from a prospective databank of consecutive patients having undergone AwFR for a low-grade glioma performed by the senior author (H.D) over a period of 26 years (1997–2023). Eligible patients met the following criteria: (1) patients with a supratentorial histo-molecularly confirmed diagnosis of IDHm-LGG according to the 2021 WHO classification^1^ (see Supplementary methods for more details regarding histomolecular data acquisitions); (2) adult patients with age ≥18 years; (3) intraoperative use of functional mapping to guide resection under awake condition (4) with at least 3 months of follow-up. Patients without information regarding the family status before/after surgery were excluded.

### Standard Protocol Approvals, Registrations, and Patient Consents

The study was approved by an independent institutional review board of the ethical comity of research from the French National College of Neurosurgery (N°00011687-2024/51). Relevant data were prospectively collected and extracted from a prospective databank (collection Neurologie DC-2013-2027). Written informed consent was obtained from the patients. Patients were not subjected to interventions outside the routine clinical management.

### Surgical Procedure

All patients were operated on with intraoperative functional mapping using direct electrostimulation (DES) under awake conditions. Methodological details of this surgical approach have been extensively reported in previous reports.^25,26^ Tumor removal has been systematically pursued up to functional boundaries with the goal of optimizing the extent of resection while preserving the critical neural networks. Details regarding intraoperative language, motor, and cognitive monitoring are presented in the Supplementary Methods.

### Data Collection

#### *Clinical outcomes*.—

The marital status was obtained during standard medical interviews before surgery and, therefore, every 6 months until the end of the follow-up or the death of the patient. Professional activities were systematically assessed during the presurgical examination and at 12 months postsurgery. Patients’ occupations were classified using the International Standard Classification of Occupations (ISCO-08), an occupational classification organizing all jobs into a defined set of groups (from 0 to 9) according to the tasks and duties undertaken in the job and their respective skill levels (“low,” “medium,” and “high”) (https://ilostat.ilo.org/methods/concepts-and-definitions/classification-occupation, International Labour Organization, United Nations agency). Adjuvant chemotherapy and radiotherapy were considered for subsequent analyses if they were delivered within 12 months following surgical resection.

#### *Radiological data*.—

T1-weighted and fluid-attenuated inversion recovery (FLAIR) MRIs were acquired systematically before and at 3 months postsurgery. Radiological measures were blindly assessed by 2 observers. Details regarding volumetric calculations are reported in the Supplementary Methods. Resections were categorized as follows, based on the 3-month postsurgery FLAIR MRI^27,28^: partial resection (postsurgical tumor volume [PostTV > 10 mL]), subtotal resection (PostTV > 0 mL and ≤ 10 mL), total resection (defined by no postsurgical tumor residue on FLAIR-weighted MRI), and supratotal resection (defined by no postsurgical tumor residue and additional resection margins beyond the presurgical pathological FLAIR signal).^29^

Cavity resections were delineated on the T1-weighted MRIs acquired at 3 months postsurgery. Tumor infiltrations were delineated on the FLAIR-weighted MRI acquired the day before surgery. To mitigate bias arising from lesion-related neuroimaging signals deformations, all images were co-registered to the Montreal Neurological Institute space using enantiomorphic normalization^30^ with the Clinical toolbox (https://www.nitrc.org/projects/clinicaltbx) and SPM12 (https://www.fil.ion.ucl.ac.uk/spm/software/spm12) implemented in the MATLAB environment (Release 2022a, The MathWorks Inc., Natick, MA, USA). Further, resection cavity maps from the initial surgery (ROI_tumor_res_) and tumor infiltration maps (ROI_tumor_) were obtained by manual delineation using MRicron software (https://www.nitrc.org/projects/mricron). Finally, supratotal resection masks (ROI_supra_tumor_res_), defined as voxels within ROI_tumor_res_ and outside ROI_tumor_ were computed using binary operations in the FMRIB Software Library program (FSL, version 6.0, https://fsl.fmrib.ox.ac.uk/fsl).

### Statistical Analysis

#### *Descriptive analyses*.—

Data were analyzed from May 2024 to August 2024. Patients’ demographic, clinical, radiological, and histomolecular characteristics were analyzed with descriptive statistics. Given the non-Gaussian distribution of variables, continuous variables were analyzed with 2-tailed nonparametric Mann–Whitney *U* tests. Categorical variables were analyzed with Fisher’s exact test and the Chi-square test when appropriate. The median follow-up was obtained with the reverse Kaplan–Meier method.

#### *Survival analyses*.—

OS was defined as the time between surgery and death. Survival analyses were first conducted with the Kaplan–Meier method, and comparisons of survival curves were performed with the log-rank test. Cox proportional hazard models (Cox-PHM) were used to investigate associations between prognostic variables and OS, in multivariable settings. The proportionality of hazards was checked by inspection of the Schoenfeld residuals and log-minus-log survival plots. The linearity assumptions were checked by inspection of the deviance residuals vs covariate graph plots. The proportional hazard assumptions were not met for various univariable and multivariable models including, among others, sex, EOR, presurgical tumor volume (PreTV), postTV, and the use of adjuvant radiotherapy. Further, to investigate the impact of variables ‘union/marriage’ vs “single” before surgery and ‘having child/children’ vs “not having child” before surgery on OS, which have been suggested by several oncological groups in high-grade gliomas, we computed a propensity score matching analysis. A caliper size of one-fourth of a standard deviation of the sample estimated propensity scores was applied. Matching was based on age, sex, 1p19q-codeletion status, preTV, postTV, tumor location, chemotherapy, radiotherapy, 3-month postsurgical KPS, and ISCO-08 skill level. Characteristics of the matched subjects were systematically reviewed for comparisons.

#### *Multiple logistic regressions*.—

Multiple logistic regressions were conducted to examine the relationship between dependent variables “separation/divorce” (model A), “having a new child or having a new partner” (model B), and predictor variables (age, sex, 1p19q-codeletion, EOR, long-term epilepsy, ISCO skill level, educational level). The linear relationship between the logit of the outcome and the continuous predictor variables was systematically checked with the Box–Tidwell procedure. Multicollinearity among the predictors was systematically checked.

#### *Lesion-symptom mapping*.—

The relationship between ROI_tumor_res_ and the binary variable ‘separation/divorce’ was further tested on a voxel-voxel basis and on an atlas basis (Automated Anatomical labeling Atlas 3 [AAL]) using Liebermeister statistical tests (Niistat, https://www.nitrc.org/plugins/mwiki/index.php/niistat), as previously published.^31^ All analyses were conducted after controlling for lesion volume by applying the ‘regress on volume’ option. Results were corrected by using multiple comparison permutation tests (*n* = 10 000), as recommended. *P* < .05 was considered indicative of a statistically significant relationship. Using the same methodology, we repeated this analysis to investigate the relationship between ROI_supra_tumor_res_ and the binary variable “separation/divorce.”

Graphical presentations were performed in Graphpad Prism 9.0 (https://www.graphpad.com) and Inkscape 1.1 (https://www.graphpad.com). All statistical analyses were conducted with R 4.3.2 (https://www.r-project.org), including the MatchIt package (https://cran.r-project.org/web/packages/MatchIt), the optmatch package (https://cran.r-project.org/web/packages/optmatch) and the lessR package (http://cran.nexr.com/web/packages/lessR).

## Results

### Cohort Description

**Figure 1.**
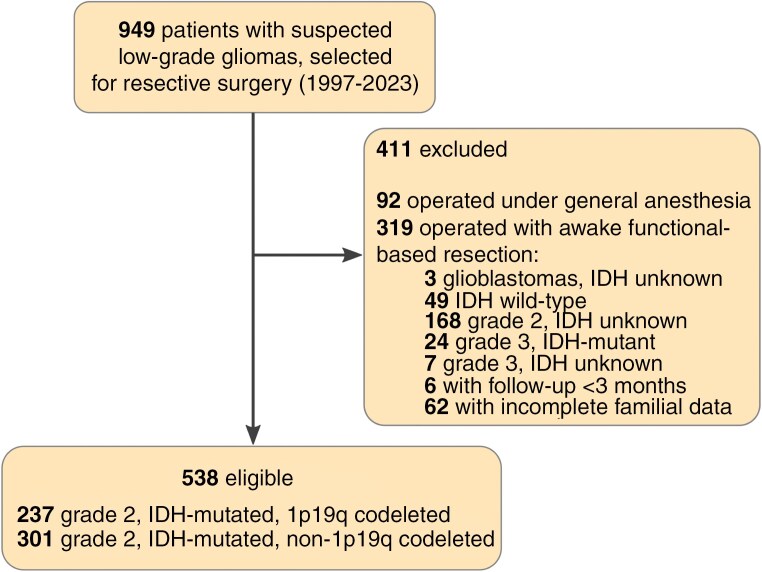
Data flow diagram. IDH indicates isocitrate dehydrogenase gene 1 or 2.

**Figure 2.**
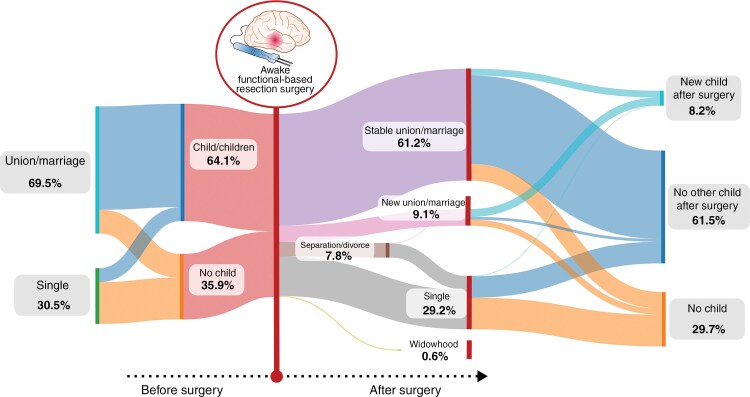
Sankey diagram (*n* = 538) illustrating long-term family trajectories. Reported percentages may not add up to 100% due to rounding. To simplify the diagram, the number of children among patients who became widows (*n* = 3, 0.6%; 1 had no children and 2 had children before surgery) has not been visually reported or included in the percentages shown.

Among the 949 patients included in the databank, 538 patients fulfilled the inclusion criteria (see flow chart, Figure 1). The cohort’s characteristics are detailed in Table 1, and a Sankey diagram provides an overview of the main family outcomes (Figure 2). The cohort included 292 males ([54.2%], mean age 37.2 ± 9.7 years, median KPS score 93.9 ± 92) who underwent AwFR for an IDHm-LGG (301 IDH-mutant grade 2 astrocytomas, 237 IDH-mutant 1p19q codeleted grade 2 oligodendrogliomas). Before surgery, 286 patients (53.2%) were married, 88 (16.3%) lived with a partner, 36 (6.7%) were divorced/separated, 2 (0.3%) were widowed and 126 (23.4%) were single. Moreover, 345 patients (64.1%) had 1 to 6 children. No patients but 4 experienced permanent postoperative deficit (0.7%). The mean KPS score was 93.9 ± 6.4 at 3 months after surgery, with 91.7% of patients who returned to work at 12 months. At the end of the follow-up, the family status was stable in 399 patients (74.2%). Among 374 patients who lived with a partner (union/marriage) before the onset of the disease, 42 patients divorced/separated (1 of them having a remarriage). Among 162 patients who were single/divorced/separated before surgery, 48 patients changed their status and presented with a union/marriage at the end of the follow-up. Finally, 3 patients who were married before surgery (1%) have become widows. Furthermore, in the full cohort, 44 patients (8.2%) had 1 child (32 [6.3%] patients) or 2 children (12 [2.2%] patients) during the follow-up. In addition, among 193 (35.9%) patients who had no children before diagnosis, 33 (17.1%) patients had a child for the first time following the onset of the disease.

**Table 1.** Characteristics of the Cohort

Variables	Overall (*n* = 538)	IDH-mutant Astrocytomas (*n* = 301)	IDH-mutant, 1p19q codeleted oligodendrogliomas (*n* = 237)	*P*-value
Age at surgery, y, median (IQR)	36.0 (30–44)	34.0 (29–41)	39 (32–47)	<.0001^a^
*Sex*				
Female, *n* (%)	246 (45.7)	136 (45.2)	110 (46.4)	.794^b^
Male, *n* (%)	292 (54.3)	165 (54.8)	127 (53.6)	
*Tumor volumes*				
Presurgical TV, mL, median (IQR)	43.0 (21.0–85.0)	50 (22.5–90)	39 (20-75)	.092^a^
Postsurgical TV, mL, median (IQR)	2.0 (0.0–8.0)	2.0 (0.0–9.5)	2.5 (0.0–6.75)	.654^a^
*Postoperative seizures*				
Long term (>3 months), *n* (%)	38 (7.1)	26 (8.6)	12 (5.1)	.128^b^
Transient (<3 months), *n* (%)	50 (9.3)	27 (9.0)	23 (9.7)	.767^b^
*Adjuvant chemotherapy*				
Yes, *n* (%)	42 (7.8)	27 (9.0)	15 (6.3)	.332^b^
No, *n* (%)	496 (92.2)	274 (91.0)	222 (93.7)	
*Adjuvant radiotherapy*				
Yes, *n* (%)	9 (1.7)	6 (2.0)	3 (1.3)	.738^b^
No, *n* (%)	529 (98.3)	295 (98.0)	234 (98.7)	
Extent of resection (median, IQR)	95.0 (88.0–100.0)	95.0 (88–100)	94.0 (87–100)	.517^a^
*Type of resection*				
Supratotal, *n* (%)	48 (8.9)	27 (9.0)	21 (8.9)	>.999^b^
Total, *n* (%)	125 (23.2)	72 (23.9)	53 (22.4)	.682^b^
Subtotal, *n* (%)	266 (49.4)	141 (46.8)	125 (55.7)	.193^b^
Partial, *n* (%)	99 (18.4)	61 (20.3)	38 (16.0)	.220^b^
Postoperative deficit, *n* (%)	4 (0.7)	2 (0.6)	2 (0.8)	>.999^b^
*Professional activities*				
Active before surgery, *n* (%)	449 (83.5)	257 (85.4)	192 (81.0)	.199^b^
Active at 12 months after surgery, *n* (%)	422 (78.4)	244 (81.2)	178 (75.1)	.113^b^
ISCO-08 Low skill level	30 (5.6)	17 (5.6)	13 (5.5)	>.999^b^
ISCO-08 Medium skill level	166 (30.9)	96 (31.9)	70 (29.5)	.574^b^
ISCO-08 high-skill level	251 (46.7)	137 (45.5)	114 (48.1)	.602^b^
Median follow-up, y (95% CI)	7.8 (7.1–8.3)	6.8 (6.1–7.5)	9.2 (8.2–9.6)	<.0001^c^

^a^Two-tailed Mann–Whitney *U* test.

^b^Fisher’s exact test.

^c^Log-rank test, reverse Kaplan–Meier method.

### Survival Analyses

**Figure 3.**
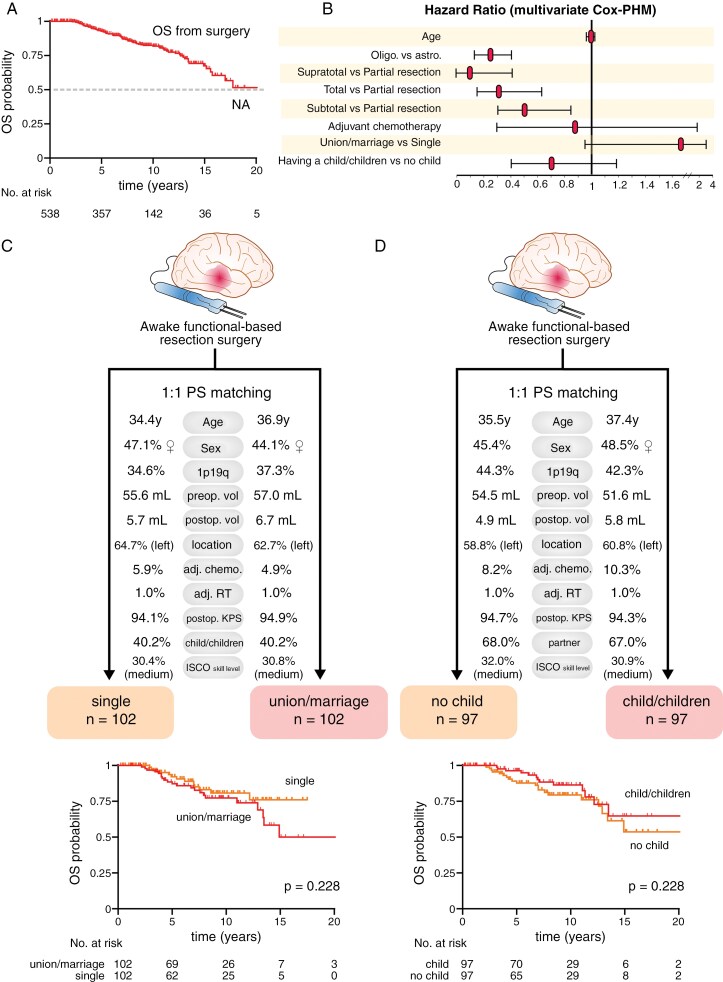
Impact of family status on overall survival. (A) Kaplan–Meier curve for overall survival (OS) from initial surgery in all patients (*n* = 538). (B) Hazard ratio results for overall survival (*n* = 538) using multivariate Cox proportional hazard models in variables eligible for analyses. (C) From top to bottom, characteristics of patients selected for comparison with propensity score matching (living alone vs. partner) and Kaplan–Meier curves for overall survival stratified by family status. Log-rank tests were used for statistical comparisons. (D) From top to bottom, characteristics of patients selected for comparison with propensity score matching (no child vs child/children) and Kaplan–Meier curves for overall survival stratified by family status. Log-rank tests were used for statistical comparisons. Cox-PHM, Cox proportional hazard model, OS, overall survival.

The median follow-up was 7.7 years (95% CI: 7.1–8.3 years, by reverse Kaplan–Meier estimation), and the median OS was over 20 years (95% CI: 15.8-NA years, Figure 3A). In Cox-PHM (Figure 3B), oligodendroglioma subgroup (multivariable HR: 0.23, 95% CI: 0.14–0.39, *P* < .0001), supratotal resection (multivariable HR: 0.09, 95% CI: 0.005–0.43, *P* = .019), total resection (multivariable HR: 0.33, 95% CI: 0.16–0.64, *P* = .001) and subtotal resections (multivariable HR: 0.49, 95% CI: 0.30–0.84, *P* = .007) were significantly associated with longer OS. Adjuvant chemotherapy did not affect OS. Family status (union/marriage or having child/children) did not affect OS. Further propensity score matching analyses confirmed that union/marriage and having a child/children did not impact OS. Characteristics of the matched cohorts and associated survival curve comparisons are illustrated in Figure 3C and D.

### Predictors of Separation/Divorce and New Union/Marriage or New Child/Children

**Figure 4.**
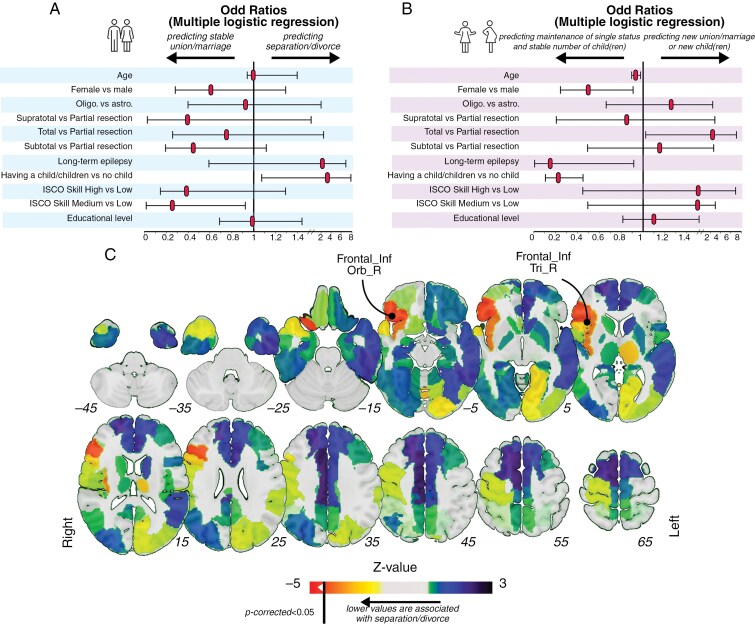
Factors associated with long-term family outcomes. (A) Relationship between separation/divorce and other oncological, professional, and clinical variables (multiple logistic regression, *n* = 538). (B) Relationship between new union/marriage or new child/children and other oncological, professional, and clinical variables (Multiple logistic regression, *n* = 538). (C) Atlas-based lesion-symptom mapping results, using “separation/divorce” as a binary variable (*n* = 488). The Automated Anatomical labeling Atlas 3 (AAL) atlas was used. Liebermeister measures were processed. Results were corrected by using multiple comparison permutation tests (*n* = 10 000). *P* < .05 was considered indicative of a statistically significant relationship.

Multiple logistic regressions were conducted to analyze the relationship between separation/divorce (model A), new union/marriage or new child/children (model B), and other oncological, professional, and clinical variables. All models’ goodness-of-fit was assessed using a Hosmer–Lemeshow test (*χ*^2^ = 9.66, *P* = .290 [model A]), (*χ*^2^ = 7.55, *P* = .479 [model B]). Occupations associated with medium skill level according to the ISCO-08 classifications predicted stable union/marriage (OR: 0.27, 95% CI: 0.05–0.95, *P* = .035, Figure 4A), while having a previous child predicted separation/divorce (OR: 2.73, 95% CI: 1.07–7.83, *P* = .045). Prolonged single status was predicted by greater age (OR: 0.94, 95% CI: 0.91–0.98, *P* = .010, Figure 4B), female gender (OR: 0.48, 95% CI: 0.25-0.91, *P* = .028), previous child/children (OR: 0.23, 95% CI: 0.11–0.45, *P* < .0001) and tended to be associated with long-term epilepsy (OR: 0.17, 95% CI: 0.009–0.95, *P* = .062). Total resection predicted new union/marriage and/or new child/children (OR: 2.59, 95% CI: 1.04–7.10, *P* = .046).

### Lesion-Symptom Mapping for “Separation/Divorce” Outcomes

Voxel-based lesion-symptom mapping (*n* = 488) revealed no significant statistical associations between ‘separation/divorce’ and ROI_tumor_res_. AAL atlas-based lesion-symptom mapping revealed that the parcels “Frontal_Inf_Orb_R” (right pars orbitalis) and ‘Frontal_Inf_Tri_R’ (right pars triangularis) were significantly associated with separation/divorce (Liebermeister measures, after controlling for lesion volume, *p*_corrected_ < .05, 10 000 permutations, Figure 4C). No statistical associations were found between “separation/divorce” and ROI_supra_tumor_res_.

## Discussion

Patients with IDHm-LGG are usually young and active at diagnosis, with no or only few functional deteriorations. Advances in neuro-oncological treatments such as early and maximal “safe” surgical resection, which has been reappraised as a major prognostic factor,^4^ have considerably improved the lifespan of glioma patients and now prompt a greater emphasis on quality of life. Strikingly, although family status is one of the cornerstones of quality of life, this critical aspect has received only limited attention in patients with IDHm-LGG.

In the present study, we leveraged a unique consecutive cohort of IDHm-LGG patients who benefited from a homogeneous AwFR surgical approach at diagnosis. We took advantage of a standardized prospective collection of data over a long period of 26 years to examine long-term familial outcomes in conjunction with known prognostic factors, including clinical characteristics, molecular findings, tumor location, and therapeutics. First, we observed that a high rate of union/marriage stability was maintained following IDHm-LGG diagnosis and AwFR. Remarkably, even though 7.8% of patients divorced/separated, 9.1% of patients had the opportunity to find a new partner. These results support the fact that few IDHm-LGG patients had an aborted union/marriage even with a long follow-up since the diagnosis of an incurable brain tumor. Conversely, these original data show that the majority of IDHm-LGG patients wanted to make long-term projects, including raising or enlarging their family with a new child/children in 8.2% of cases.

Several predictive factors of family outcomes were highlighted. Prolonged single status and absence of new child/children were predicted by age, female gender, and previous child/children. Collectively, these findings may reflect the psychological and oncological challenges faced by women with IDHm-LGG who have a desire for motherhood. It is now well-established that pregnancy can cause measurable changes in glioma behavior.^19^ Although these findings are still debated,^32^ a recent study reported an increased risk of malignant transformation following pregnancy, which may directly impact OS, especially when the tumor velocity expansion is not stable or when complete resection is not achievable.^20^

As expected, the presence of long-term epilepsy tended to predict poor family outcomes, which is congruent with the known effect of epilepsy on the quality of life of patients with brain tumors.^33^ Importantly, gross total resection (complete removal of the FLAIR MRI signal) was associated with new union/marriage and/or new child/children. This finding suggests that gross total resection may be associated with more sustainable personal and family projects. Such long-term quality-of-life benefits must be interpreted in light of the reported advantage of gross total resection in terms of OS^4,34^ and seizure control^35,36^ in patients who may be free of oncological therapies in the first years following surgery.^37^ Besides these clinical and therapeutic factors, the occupational skill level (ISCO-08) was also identified as a significant predictor of stable union/marriage. Furthermore, lesion-symptom mapping analyses examining the relationship between ROI_tum_res_ and the rate of separation/divorce unveiled a potential contribution of the right inferior frontal gyrus to the stability of the family status, which may coincide with the participation of the right pars orbitalis and triangularis to emotion recognition / mentalizing networks.^17,38,39^ Such neuroanatomical findings must be interpreted with great caution, as our results highlight that there are no absolute predictors of family outcomes. Instead, a high level of interaction exists between biological (anatomical), therapeutic, clinical, and socio-professional factors. Yet, these data prompt neurosurgeons to actively develop intraoperative awake monitoring protocols for higher-order cognitive functions in the right “non-dominant” hemisphere.^40,41^ Although we did not objectify a similar statistical association when considering only voxels resected outside of FLAIR infiltration (ROI _supra_tumor_res_), these results also question the feasibility of supratotal resection in the right inferior frontal gyrus without appropriate intraoperative monitoring, especially in view of recent research indicating that neurocognitive resilience inside and outside FLAIR infiltration follows a different degree of functional reorganization in specific anatomical settings.^42^

### Limitations

This study has several limitations. First, participants selected for retrospective analysis presented with resectable IDHm-LGGs (as determined by the treating neurosurgeon). Consequently, our findings cannot be generalized to patients with nonresectable IDHm-LGGs. Second, changes in long-term family outcomes are multifactorial in nature, and the present study could not capture exhaustively the economic, cultural, or social variables that could plausibly impact patients’ family choices. However, it should be noted that significant efforts were made to investigate a wide range of determinants, including occupational, clinical, biological, and therapeutic factors. Third, the noncomparative nature of our study prevents us from drawing direct conclusions about the specific impact of surgery and/or awake functional-guided resection on family outcomes. To the best of the authors’ knowledge, however, there is no available comparative cohort of IDHm low-grade glioma patients treated under general anesthesia or who underwent biopsy that would allow for a comparison of long-term family outcomes. Additionally, comparisons with the general population would require dedicated epidemiological studies and/or population-based analyses, which are beyond the scope of the current study. Indeed, there are several limitations when attempting to compare our results with population-based statistics. These include key differences in population characteristics, variability in sociodemographic and cultural factors across countries (as a significant proportion of patients in this series originated from different countries), and the extended recruitment period spanning over 26 years (during which marriage and divorce rates have changed significantly). Moreover, official statistics in France reflect only divorce rates and do not capture self-declared separations—an important consideration given the substantial decline in marriage rates in Western countries, including France. Nevertheless, based on publicly available data from the Institut National de la Statistique et des Études Économiques (INSEE, https://www.insee.fr/fr/statistiques/7624542?sommaire=7624746), we estimated that the average divorce rate within 0–10 years of marriage for unions formed between 1997 and 2016 (the most recent data available) ranges from 17.1% (for couples married in 1997) to 20.5% (for those married in 2016). These data suggest that the divorce rate in the general population may be higher than the divorce or separation rate observed in our study.

## Conclusion

Taken together, these findings offer a unique overview of the long-term family outcomes in IDH-mutated low-grade glioma patients who benefited from awake functional-based resection surgery. Most patients presented with a stable family status or continued to have family projects despite the onset of the disease. Critical interactions between clinical, familial, socio-professional, biological, and oncological factors were highlighted. This information may be of the utmost importance in providing relevant counsel to patients who have been diagnosed with an IDH-mutated low-grade glioma and to orient personalized follow-up and oncological management.

## Supplementary Material

vdaf102_suppl_Supplementary_Materials

## Data Availability

Data will be shared upon request to the corresponding author.
