# Supplementary material for: Long-term family outcomes in grade 2 IDH-mutated glioma patients treated with awake-guided surgery: Biological, professional, and therapeutic interactions
Source: Neurooncol Adv. 2025 May 20;7(1):vdaf102. doi: 10.1093/noajnl/vdaf102 (PMC12202148; doi:10.1093/noajnl/vdaf102)
Supplement: vdaf102_suppl_Supplementary_Materials [file vdaf102_suppl_supplementary_materials.docx]

Supplementary Materials

**Long-term family outcomes in grade 2 IDH-mutated glioma patients treated with awake-guided surgery: biological, professional, and therapeutic interactions.**

Sam Ng and Hugues Duffau

**Affiliations:**

Department of Neurosurgery, Gui de Chauliac Hospital, Montpellier University Medical Center, Montpellier, France (SN, HD); Institute of Functional Genomics, University of Montpellier, INSERM, CNRS, Team “Plasticity of Central Nervous System, Stem Cells and Glial Tumors,” National Institute for Health and Medical Research (INSERM), U1191 Laboratory, Montpellier, France (SN, HD)

**Running title:** Family status and low-grade glioma surgery

**Corresponding author**

Dr Sam Ng, M.D,

Department of Neurosurgery, Gui de Chauliac Hospital, Montpellier University Medical Center, 80 Avenue Augustin Fliche, 34295 Montpellier, France

Phone: +33 4 67 33 66 12; Fax: +33 4 67 33 69 12; Email: [s-ng@chu-montpellier.fr](mailto:s-ng@chu-montpellier.fr)

**Supplementary Methods 1.** Details on histomolecular acquisitions.

**Supplementary Methods 2.** Details on awake functional-based resection (AwFR) technique.

**Supplementary Methods 3**. Details on volumetric measures.

**Supplementary table 1**. Details on professional activities before/after surgery

**Supplementary table 2**. Survival results (univariable and multivariable Cox proportional hazard models)

**Supplemental figure 1:** Progression-free survival results

**Supplementary Methods 1.** Details on histomolecular acquisitions.

Immunohistochemistry for IDH1 R132H was systematically used. Between 2010 and 2018, the determination of IDH gene mutation status was made by direct Sanger sequencing. Since 2018, mutations in IDH genes have been sought as part of a wider panel of next-generation sequencing (NGS) analyzed genes (French Institut National du Cancer “INCa” panel, second edition between 2018 and 2019, then third edition since 2019).

The 1p19q codeletion has been systematically investigated in low-grade gliomas since 2007. From 2007 to 2017 a Loss-of-heterozygosity assay (LOH) with 12 microsatellite markers was performed. Since 2017 all Copy Number Variations (CNVs) have been analyzed by array comparative genomic hybridization (a-CGH)

Of note, among 538 patients with histomolecular data included in the study, some patients operated on before 2009 presented with incomplete molecular data (especially regarding 1p19q co-deletion information). All were re-operated during the follow-up and finally included in the study based on updated molecular findings obtained during the second tissue sampling molecular analysis.

**Supplementary Methods 2**. Details on awake functional-based resection (AwFR) technique

The same neurosurgeon (BLINDED) performed the same surgical technique on all patients selected for analyses. Intrasurgical electrical mapping was performed through cortical and subcortical direct electrostimulation (DES) using an asleep-awake-asleep protocol.

Briefly, following wide craniotomy under general anesthesia, the cortical surface was exposed, and the tumor was visualized by intraoperative ultrasound. Once the patient was awake, electrical mapping was achieved with a bipolar electrode probe with a 5 mm inter-tip spacing (NIMBUS Stimulator, Newmedic, France), delivering a biphasic electric current (60 Hz, 1 ms pulse width, amplitude 1.50 to 3.50 mA). The amplitude was progressively increased until a transient speech arrest response from the ventral premotor cortex was attained, according to the Ojemann method.^1^ This amplitude was not modified during the remainder of the intrasurgical electrical mapping (including both cortical and subcortical axonal mapping). After completion of the cortical mapping, the tumor removal was performed by subpial dissection. Subcortical white matter DES mapping was performed to achieve tumor resection according to individual functional boundaries. Importantly, a stimulation site was considered functional if DES elicited disturbances at least three times in a nonconsecutive manner. Intraoperative monitoring of language, motor, and cognitive functions was achieved by a senior speech therapist and/or neuropsychologist who remained blinded to DES application.

The following tasks could be used, depending on the location of the tumor: motor tasks,^2^ a picture naming task,^3^ a semantic association task (Pyramids and Palm Trees test),^4^ a dual-task (picture naming plus movement),^5^ reading tasks,^6^ a mentalizing task (adapted version of the Read the mind in the eyes),^7^ a visual field monitoring tasks,^8^ a line bisection task,^9^ and a self-evaluation task.^10^

1. Whitaker HA, Ojemann GA. Graded localisation of naming from electrical stimulation mapping of left cerebral cortex. *Nature*. 1977;270(5632):50-51. doi:10.1038/270050a0

2. Rech F, Herbet G, Gaudeau Y, et al. A probabilistic map of negative motor areas of the upper limb and face: a brain stimulation study. *Brain*. 2019;142(4):952-965. doi:10.1093/brain/awz021

3. Herbet G, Moritz-Gasser S, Boiseau M, Duvaux S, Cochereau J, Duffau H. Converging evidence for a cortico-subcortical network mediating lexical retrieval. *Brain*. 2016;139(11):3007-3021. doi:10.1093/brain/aww220

4. Moritz-Gasser S, Herbet G, Duffau H. Mapping the connectivity underlying multimodal (verbal and non-verbal) semantic processing: A brain electrostimulation study. *Neuropsychologia*. 2013;51(10):1814-1822. doi:10.1016/j.neuropsychologia.2013.06.007

5. Duffau H, Ng S, Lemaitre AL, Moritz-Gasser S, Herbet G. Constant Multi-Tasking With Time Constraint to Preserve Across-Network Dynamics Throughout Awake Surgery for Low-Grade Glioma: A Necessary Step to Enable Patients Resuming an Active Life. *Front Oncol*. 2022;12:924762. doi:10.3389/fonc.2022.924762

6. Ng S, Moritz-Gasser S, Lemaitre AL, Duffau H, Herbet G. White matter disconnectivity fingerprints causally linked to dissociated forms of alexia. *Commun Biol*. 2021;4(1):1413. doi:10.1038/s42003-021-02943-z

7. Yordanova YN, Cochereau J, Duffau H, Herbet G. Combining resting state functional MRI with intraoperative cortical stimulation to map the mentalizing network. *NeuroImage*. 2019;186:628-636. doi:10.1016/j.neuroimage.2018.11.046

8. Gras-Combe G, Moritz-Gasser S, Herbet G, Duffau H. Intraoperative subcortical electrical mapping of optic radiations in awake surgery for glioma involving visual pathways. *JNS*. 2012;117(3):466-473. doi:10.3171/2012.6.JNS111981

9. de Schotten MT, Urbanski M, Duffau H, et al. Direct Evidence for a Parietal-Frontal Pathway Subserving Spatial Awareness in Humans. *Science*. 2005;309(5744):2226-2228. doi:10.1126/science.1116251

10. Ng S, Herbet G, Lemaitre AL, Moritz-Gasser S, Duffau H. Disrupting self-evaluative processing with electrostimulation mapping during awake brain surgery. *Sci Rep*. 2021;11(1):9386. doi:10.1038/s41598-021-88916-y

**Supplementary Methods 3.** Details on volumetric measures

Tumor volumes were obtained on FLAIR-weighted MRI by manual segmentation, by two blinded observers:

-In the first period of the series (1997-2008, n=50 patients), an estimation of these volumes was obtained by the ellipsoid approximation (D1×D2×D3/2).

-Since 2008, a dedicated software (Myrian, Intrasense, France) has been used for segmentation.

Presurgical tumor volumes were obtained on the presurgical MRIs performed 24-72h before surgery. Postsurgical tumor volumes were computed on the 3-month postoperative MRIs to avoid artifacts related to blood signals and tissue deformations.

**Supplementary table 1**. Details on professional activities before/after surgery

| **Professional activities** |  |
| --- | --- |
| **Before surgery** |  |
| Active/ Employed before surgery, n (%) | 449 (83.5) |
| Unemployed before surgery, n (%) | 81 (15.1) |
| Retired before surgery, n (%) | 8 (1.5) |
| **12 months after surgery** |  |
| Active/ Employed after surgery, n (%) | 422 (78.4) |
| Unemployed after surgery, n (%) | 108 (20.1) |
| Employed before and unemployed after surgery, n (%) | 46 (10.2) |
| Unemployed before and employed after surgery, n (%) | 19 (22.2) |
|  |  |

**Supplementary table 2**. Main family outcomes (n=538)

|  |  |
| --- | --- |
| **Before surgery** |  |
| Union/marriage, n (%) | 374 (69.5) |
| Celibacy, n (%) | 164 (30.5) |
| Child/children, n (%) | 345 (64.1) |
| No child, n (%) | 193 (35.9) |
| **End of follow-up** |  |
| Stable union/marriage, n (%) | (61.2) |
| Separation/divorce, n (%) | 41 (7.6) |
| Celibacy, n (%) | 157 (29.2) |
| Widowhood, n (%) | 3 (0.6) |
| New child, n (%) | 44 (8.2) |
| No other child, n (%) | 331 (61.5) |
| Never had child, n (%) | 160 (29.7) |
